# Emergency Department Overcrowding Despite Lower Post-Pandemic Volumes: A Multicenter Study

**DOI:** 10.3390/jcm15197450

**Published:** 2026-09-24

**Authors:** Paolo Bima, Giovenale Moirano, Elvira Fanelli, Giulia Colombo, Marcello Candelli, Maurizio Berardino, Franco Aprà, Giorgio Costantino, Francesco Franceschi, Stefano Roggero, Antonio Scarmozzino, Enrico Lupia, Milena Maule, Fulvio Morello

**Affiliations:** 1 Dipartimento di Scienze Mediche, Università degli Studi di Torino, 10126 Turin, Italy; 2 S.C. Epidemiologia dei Tumori U, A.O.U. Città della Salute e della Scienza di Torino, 10126 Turin, Italy; 3 Medicina e Chirurgia d’Accettazione e d’Urgenza, Ospedale San Giovanni Bosco, 10154 Turin, Italy; 4 Fondazione IRCCS Ca’ Granda Ospedale Maggiore Policlinico, UOC Pronto Soccorso, 20122 Milan, Italy; 5 Dipartimento Scienze Emergenza, Anestesiologiche, Rianimazione, Università Cattolica Sacro Cuore, Fondazione Policlinico Universitario A. Gemelli IRCCS, 00168 Rome, Italy; 6 Dipartimento di Anestesia, Rianimazione ed Emergenza, Ospedale CTO, A.O.U. Città della Salute e della Scienza di Torino, 10126 Turin, Italy; 7 Dipartimento di Scienze Cliniche e di Comunità, Università degli Studi di Milano, 20122 Milan, Italy; 8 Direzione Sanitaria Presidio Molinette, A.O.U. Città della Salute e della Scienza di Torino, 10126 Turin, Italy; 9 S.C. Medicina d’Urgenza U, Ospedale Molinette, A.O.U. Città della Salute e della Scienza di Torino, 10126 Turin, Italy

**Keywords:** emergency department, visits, crowding, boarding, COVID-19

## Abstract

**Background**: The COVID-19 pandemic profoundly affected emergency department (ED) activity worldwide, and health system constraints may persist beyond the pandemic phase. **Methods**: This multicenter retrospective study included all ED visits by patients aged ≥14 years at four Italian tertiary EDs in 2019 and 2024. Input, throughput, and output measures were compared using negative binomial regression and Weibull accelerated failure time models. **Results**: Overall, 286,298 visits occurred in 2024 and 310,900 in 2019 (adjusted change, −5.5%; 95% CI −9.8 to −1.0). The reduction mainly involved low-priority and daytime presentations. Despite lower input, flow metrics and crowding worsened. Door-to-physician time increased from 51 to 58 min (+21.6%; 95% CI 20.9 to 22.4). Median ED occupancy increased by 8.0% (95% CI 4.5 to 11.6), and the number of patients who left without being seen (LWBS) increased by 17.1% (95% CI 12.9 to 21.5). Total ED LOS increased from 3 h 48 min to 4 h 27 min (+15.1%; 95% CI 14.4 to 15.7). The number of admitted patients with an ED LOS of ≥12 h increased by 13.8% (95% CI 10.3 to 17.3), while ED LOS among admitted patients increased from 13 h 17 min to 19 h 21 min (+29.0%; 95% CI 27.3 to 30.8). ED LOS increased most for admissions to medical wards but decreased for critical care admissions. **Conclusions**: In 2024, ED volumes were lower than in 2019, whereas ED occupancy, LWBS, and ED LOS were higher, with substantial heterogeneity across centers. These findings suggest a role for hospital output constraints, but the descriptive design does not establish causality.

## 1. Introduction

Emergency department (ED) overcrowding occurs when demand for emergency care exceeds the available resources and is a longstanding global problem [1,2]. Crowding arises from a variety of factors, including increased patient influx, staffing limitations, prolonged boarding times, and broader system-level constraints [3]. Regardless of its causes, ED overcrowding has consistently been associated with adverse consequences for both patients and healthcare staff. Specifically, it has been linked to increased in-hospital mortality, delays in the treatment of acute pain, and reduced adherence to guideline-directed care [4,5,6]. In addition, prolonged waiting times contribute to a higher proportion of patients leaving without being seen (LWBS) and patients staying overnight in the ED (negatively impacting on their prognosis), while also exacerbating staff burnout and turnover [4,7,8].

Measures of ED overcrowding are commonly classified into patient flow (e.g., boarding time, LWBS proportion) and non-flow (e.g., ED crowding indexes, staff-to-patient ratio) metrics, and can be further categorized according to the phase of emergency care they assess: input, throughput, and output [3]. Input reflects patient demand; throughput concerns processes within the ED, including staffing and physical capacity; output reflects barriers to transferring patients out of the ED, such as inpatient bed availability and hospital discharge processes [9].

The onset of the COVID-19 pandemic in 2020 exposed significant vulnerabilities in EDs and healthcare systems worldwide. Total ED visits declined sharply during the initial wave, followed by a gradual but incomplete recovery in subsequent years [10,11,12,13,14]. Recent data from the United States indicate that post-pandemic ED length of stay (LOS) and boarding have increased, alongside a rise in the proportion of LWBS [2,15]. Although a similar pattern may also be occurring in Europe, the extent of post-pandemic changes in ED overcrowding and their underlying determinants remain unclear [16]. Tertiary EDs may be particularly prone to persistent post-pandemic crowding because, serving as both direct-access and referral hubs, they rely heavily on timely access to hospital resources, specialty services and inpatient beds. Herein, we sought to analyze process-level metrics of ED overcrowding in tertiary EDs across three different Italian regions, aiming to provide actionable insights for guiding physicians and public health authorities [6,17].

## 2. Materials and Methods

### 2.1. Study Setting and Design

This was a multicenter retrospective study conducted in four Italian tertiary EDs that routinely care for patients aged ≥14 years, from three different regions (Piemonte, Lombardia and Lazio). These centers are characterized by 24/7 access to specialized care including specialized intensive care units (ICUs), neuro-trauma and cardiothoracic units, interventional cardiology, and interventional radiology. Three EDs are located in academic centers (Città della Salute e della Scienza [CDSS], Turin; Ospedale Maggiore Policlinico [OMP], Milan; Policlinico Universitario Gemelli [PUG], Rome), and one in a non-academic hospital (Ospedale San Giovanni Bosco [OSGB], Turin). The CDSS unit is composed of two interconnected EDs, one functioning as a general medical and surgical ED, and one dedicated to trauma and orthopedic emergencies. The other centers operate general EDs.

### 2.2. Inclusion Criteria, Data Extraction and Aggregation

All ED visits by patients aged ≥14 years presenting to the participating EDs between 1 January and 31 December of 2019 or 2024 were included. Visits were excluded if the main reason for ED visit was pregnancy or labor, because not all participating centers managed these conditions. Data were extracted from the electronic health-record systems using site-specific automated queries. Before data extraction, the participating centers agreed upon a common set of variable definitions, formats, and inclusion criteria. Each center mapped its local data to this common structure. All hospital information systems used in the participating centers could generate structured reports containing sex, age, key clinical timestamps, triage information, final diagnosis, and disposition decision. ED records that were canceled after initial registration and before triage, as well as records without a valid triage time, were excluded. The extracted variables were date and time of ED registration and triage, triage priority, main presenting symptom recorded at triage, date and time of the first physician assessment, date and time of ED discharge, patient disposition, and final diagnosis. Data extraction did not involve individual medical-chart review or the collection of additional personal or clinical information. Before the datasets were merged, the required variables were checked for completeness and consistency with the shared definitions. Triage-related variables were subsequently harmonized across centers according to predefined common categories.

The triage priority variable was based on the initial priority code assigned by an ED triage nurse according to local protocols. The triage system changed from four to five priority levels between the two study periods. Because a reliable crosswalk across all priority levels was not available, only the lowest- and highest-priority categories were analyzed, as their intended clinical meaning remained comparable across the two systems. For main triage symptoms, we extracted the main triage symptom as flagged by the triage nurse. When “other” was selected, the triage notes were searched using predefined free-text terms. We extracted the following triage symptoms, as they were consistent across all included centers: chest pain, dyspnea, abdominal pain, trauma, intoxication/drug use, and behavioral/psychiatric problem. The main final diagnosis of each patient was defined by the ICD-9-CM code attributed at patient discharge by the attending physicians. In further analyses, final diagnoses were grouped into diagnostic categories following standard ICD-9-CM classification, as shown in Supplemental Table S1. ED disposition was classified as hospital admission, discharge home, LWBS, or death.

The source dataset contained one row per ED visit. For the analysis of count outcomes, data were aggregated as weekly counts by center and, where applicable, by sex, age category, and diagnosis group. Time-related outcomes were analyzed at the individual-visit level. ED occupancy was derived from hourly measurements of the number of patients simultaneously present in each ED and was summarized as the weekly median for each center. Reattendances were not excluded, as repeat visits by the same patient could not be identified due to the anonymized nature of the dataset.

### 2.3. Outcome Measures

The study aimed to compare adjusted differences in ED flow measures between 2019 and 2024 across the participating centers. The year 2019 was selected as the last complete calendar year before the COVID-19 pandemic, whereas 2024 was the first full calendar year after the WHO determined on 5 May 2023 that COVID-19 no longer constituted a Public Health Emergency of International Concern. Accordingly, 2019 and 2024 are referred to as the pre-pandemic and post-pandemic study years, respectively. The intervening years were not included because they represented heterogeneous pandemic and transitional phases characterized by substantial changes in COVID-19 burden and healthcare organization. The study was not designed to characterize the temporal trajectory between the two selected years.

ED input measures were total ED visits, stratified by patient age, sex, day vs. nighttime presentation (nighttime defined as 8 p.m. to 8 a.m.), triage codes, final disposition and grouped final diagnoses. ED throughput measures included door-to-physician time, number of patients who left without being seen (LWBS), and ED occupancy. Door-to-physician time was calculated as the time between triage and the first physician assessment. ED occupancy was measured as the number of patients simultaneously present in each ED at hourly intervals. For descriptive presentation, these hourly observations were summarized as the weekly median ED occupancy for each center.

ED output measures included the numbers of patients discharged, admitted, or deceased; ED LOS was stratified by final disposition and the number of admitted patients with an ED LOS of ≥12 h; ED LOS among admitted patients was stratified by destination ward. ED LOS was calculated as the interval from triage to ED discharge, as recorded in the electronic health record. True boarding time is defined as the interval between the admission decision and transfer to an inpatient bed. Because the admission-decision timestamp was not consistently available, true boarding time could not be calculated. We therefore used the number of admitted patients with an ED LOS of ≥12 h as a pragmatic proxy for prolonged boarding. The 12 h threshold was selected to identify markedly prolonged ED stays among admitted patients and was not intended as a validated definition of boarding.

Hospital beds were classified according to ward type as follows: general and specialty medicine (including internal medicine, geriatrics, general cardiology, dermatology, endocrinology, gastroenterology, hematology, infectious diseases, clinical nutrition, oncology, nephrology, neurology, and rheumatology), critical care (broadly defined to include general and specialty intensive care, coronary care, stroke, semi-intensive care or high dependency, and emergency medicine units), psychiatry, and surgery. Center-level bed capacity in general and specialty medical wards and critical care units was obtained for 2019 and 2024. Only routinely available, designated inpatient beds were counted. When bed availability varied during the year, a time-weighted annual average was calculated and rounded to the nearest whole bed. Inpatient LOS was available for two centers (CDSS, OSGB) and was calculated as the time (in days) elapsed from ED discharge (admission to hospital ward) to discharge from hospital.

### 2.4. Statistical Analysis

Counts were expressed as total number, while time variables were expressed as median. Throughout the study, the main comparison variable was defined as year 2024 versus 2019 (reference). Temporal patterns in weekly counts and time-related outcomes were visualized using time-series plots with a Locally Estimated Scatterplot Smoothing (LOESS) curve and its 95% confidence band. Overdispersion was assessed using the Cameron–Trivedi test and was detected for all count outcomes. We therefore fitted multivariable negative binomial regression models and estimated percent differences and their 95% confidence intervals from the exponentiated regression coefficients. Models were adjusted for center, sex, age category, and seasonality using a sine–cosine pair. Diagnosis group was included when applicable but was not included when diagnostic category itself was the outcome. We did not account for weekly autocorrelation, nor did we calculate robust standard errors. For time variables (door-to-physician time, ED LOS, inpatient LOS), we applied a time-to-event analysis, by fitting a multivariable parametric survival regression assuming a Weibull distribution and parameterized as an accelerated failure time model. We adjusted for center, sex, age category, diagnosis group and seasonality. Percentage changes and 95% CI were calculated from the exponentiated regression coefficients.

Subgroup analyses by age categories, sex, time of day, and triage priority were carried out to assess the consistency of the effect in different patient groups. A sensitivity analysis considering only patients aged ≥18 years was also performed.

To assess whether the adjusted differences between 2019 and 2024 varied across centers, center-by-year interaction terms were added to the models and tested using likelihood-ratio tests. All statistical tests were two-sided. All statistical analyses were performed using R version 4.5.0 (R Foundation for Statistical Computing, Vienna, Austria).

The authors used ChatGPT 5.6 Sol (OpenAI, San Francisco, CA, USA) for language checking.

## 3. Results

### 3.1. Input

The changes in input measures are summarized in Table 1. Total ED visits decreased by 5.5% in 2024 vs. 2019. In both years, weekly ED visits declined from January to December (Figure 1A). Female patients were 47% in 2024 vs. 48% in 2019, and the median age was 54 (36–72) vs. 53 (35–72) years. Patients aged 14–17 years accounted for 2.4% of the visits in 2024 and 2.7% in 2019. The reduction remained consistent after restricting the analysis to patients aged ≥18 years, across sex (Figure 1B,C) and age groups, being lowest for patients aged 50–74 years (Figure 1D–F). Three centers experienced a reduction in ED visits: −7% for CDSS, −14.9% for PUG and −15.6% for OMP, accounting for 77.1% of the visits. Only one center (OSGB) showed a +4.9% change in ED visits (Supplemental Figure S1). The reduction in ED visits was not statistically significant during nighttime (Supplemental Figure S2).

Lowest-priority visits decreased by 29.5%, whereas highest-priority visits did not change significantly (+3.1%; 95% CI −0.8 to 7.2; Figure 1G,H). Interestingly, lowest triage codes peaked in the summertime, while highest triage codes were at nadir in the same period. Among main triage symptoms in 2024, visits for behavioral/psychiatric symptoms were markedly increased (38.9%); visits for chest pain (+5.5%), dyspnea (+7.3%), and intoxication (10.7%) slightly increased (Supplemental Figure S3). Dyspnea, chest pain and trauma were lowest during summertime.

### 3.2. Throughput and Crowding

Median door-to-physician time (Figure 2A) increased from 51 min in 2019 to 58 min in 2024 (adjusted change of 21.6%; 95% CI 20.9 to 22.4), with no clear seasonal pattern. Median ED occupancy, used as an operational proxy for crowding, increased in 2024 (Figure 2B), with an adjusted change of 8.0% (95% CI 4.5 to 11.6). As an indirect measure of crowding, the number of LWBS visits increased by 17.1% (95% CI 12.9 to 21.5, Figure 2C). While median ED occupancy oscillated throughout the year, LWBS visits were less frequent during summer.

### 3.3. Output and Boarding

The changes in output measures are summarized in Table 2. Total ED LOS showed a 15.1% increase in 2024, persistent throughout the year (Figure 3).

The increase was greatest in patients eventually admitted to hospital (29%), and lower yet significant for patients ultimately discharged home (6.0%), despite a significant decrease in ED visits terminating with both home discharge and hospital admission (Figure 4A–D).

Admitted patients with an ED LOS of ≥12 h increased by 13.8% (Supplemental Figure S4). Accordingly, median ED LOS for admitted patients increased by more than 6 h in 2024 (29%). The number of patients who died in the ED also increased by 23.3% (Figure 4E). Both hospital admissions and ED deaths were less frequent during summer, while ED LOS was stable throughout the year.

### 3.4. Final Diagnoses and Hospital Admissions

Among final ED diagnoses in all patients, those related to infectious diseases and neurology increased in 2024. Those related to oncology, endocrinology, drug use and syncope were unchanged, while all others were reduced (Figure 5A). In patients admitted to hospital, diagnoses related to pneumology, neurology, nephrology, urology and gastroenterology increased in 2024, while those related to cardiovascular medicine, syncope and unspecific chest pain were reduced (Figure 5B).

The changes in output measures related to hospital admission are summarized in Table 3. Patients admitted to general and specialty medicine wards were not significantly increased, but median ED LOS among patients admitted to these wards increased by 35.8% in 2024 (Supplemental Figure S5A). Patients admitted to critical care wards decreased by 7.3% and their median ED LOS decreased by 7.5% in 2024 (Supplemental Figure S5B). Median ED LOS among patients admitted to psychiatric wards increased by 39.5% in 2024, despite a stable number of admitted patients.

For general and specialty medical wards, overall bed capacity across participating centers was 1167 beds in 2024 compared with 1188 in 2019 (absolute change, −21 beds). At the center level, bed capacity in 2024 was lower than in 2019 at two centers, unchanged at one, and higher at one (Supplemental Table S2). For critical care units, overall bed capacity was 304 beds in 2024 compared with 296 in 2019 (absolute change, +8 beds). At the center level, bed capacity in 2024 was higher than in 2019 at one center and unchanged at the other three. The median inpatient LOS, available for two centers (CDSS and OSGB), was 9.1 days in 2024 vs. 8.3 days in 2019 (+5.9%; Supplemental Table S3).

A summary of center-stratified outcomes is presented in Supplemental Figure S6. The adjusted differences between 2019 and 2024 varied across centers for all evaluated outcomes (*p* for interaction < 0.001 for all except for patients deceased in the ED: *p* = 0.03).

## 4. Discussion

This multicenter study compared ED volumes and key patient-flow metrics across four Italian tertiary EDs in 2019 and 2024. ED volumes were approximately 6% lower in 2024, mainly because of fewer low-priority and daytime presentations. This reduction was consistent across sex and age categories, but it was limited to low-priority triage codes and to daytime hours. This is consistent with a recent study conducted in the Lombardy region [16]. This selective decrease in low-priority ED visits might reflect persistent changes in healthcare-seeking behavior following the pandemic, as well as greater use of alternative care pathways by some patients, potentially influenced by prolonged ED waiting times [10,12,13,18]. Accordingly, we observed a reduction in patients discharged home from the ED, and to a lesser extent, a reduction in patients admitted to hospital, whereas admissions to general and specialty medicine were essentially unchanged. Also, the patient case-mix changed, with potential downstream effect on ED LOS due to increased complexity.

A key finding was that, despite lower ED input, crowding measures were consistently higher in 2024. Median door-to-physician time increased by approximately 22%; median ED occupancy increased by approximately 8%, LWBS visits by approximately 17% and patients who died in the ED by approximately 23%, even after adjustment for various measured covariates. This apparently paradoxical trend should be interpreted alongside the observed increase in total ED LOS (approximately 15% overall) and ED LOS for admitted patients (29%), highest among patients admitted to general and specialty medicine wards (36%) and psychiatry (40%). In contrast, time for admission to wards for critically ill patients decreased, in parallel with the decrease in the number of total admissions to these wards, suggesting that post-pandemic delays were concentrated among non-critical admissions. However, the results of this study do not imply causality and should be interpreted only as associations, given the observational nature of the study and the limited number of covariates that could be adjusted for (e.g., illness severity, comorbidities, staffing levels, and hospital occupancy rate were unavailable). In particular, because patient-level information and causes of death were unavailable, the observed difference in ED deaths cannot be interpreted as a consequence of crowding.

Our findings are consistent with a large US study on ED volumes and crowding, which showed that despite persistently lower ED volumes, median ED LOS increased by 32% for admitted patients, and even more for psychiatric patients [2]. These trends have been attributed to strained hospital capacity and limited inpatient bed availability, leading to bottlenecks and increased ED boarding, as well as to ED understaffing, which reduced operational capacity. Accordingly, inpatient capacity could be improved by prioritizing early and weekend discharges, and by developing outpatient pathways to efficiently redirect patients who can be managed as outpatients after a short ED treatment [1]. Previous studies have shown that prolonged ED boarding not only adversely affects the safety of boarded patients but also impacts those ultimately discharged home, increasing their ED LOS and the risk of clinical errors [4,19,20,21]. A crowded ED environment could be particularly deleterious for elderly patients [22,23]. However, this was not directly measured in our study.

Taken together, the longer ED LOS, the higher number of admitted patients with an ED LOS of ≥12 h, the longer inpatient LOS observed in the two centers for which these data were available, and the modest overall reduction in general and specialty medical bed capacity are compatible with hospital output constraints contributing to the observed worsening of ED crowding in 2024. However, the mechanisms underlying these findings are likely multifactorial. The observed changes in diagnostic distribution suggest that differences in patient case-mix may have contributed to longer ED stays, which were particularly evident among patients admitted to general and specialty medical wards. Other potentially relevant mechanisms—including differences in illness severity and comorbidities, ED staffing, inpatient bed occupancy, discharge processes, admission thresholds, access to post-acute care, and diagnostic or treatment pathways—were not directly or comprehensively assessed. Hospital output constraints should therefore be considered a plausible explanation rather than an established primary mechanism, and the relative contributions of these factors cannot be determined from this study.

The strengths of our study include its multicenter design, which captures regional differences in healthcare organizations and case-mix, and the use of multivariable modeling. However, several limitations should be acknowledged. First, this was a retrospective study based on administrative and electronic health record data, without individual chart review. Therefore, misclassification of chief presenting complaint and exact final diagnosis, especially in complex cases and comorbid patients, cannot be excluded. Since final diagnoses were based on ICD-9-CM codes assigned at ED discharge, these may have been influenced by local coding practices. Moreover, the triage system changed between the two study periods. Although the analysis was restricted to the lowest- and highest-priority categories, changes in triage procedures, staff training or composition, local coding practices, and thresholds for assigning priority may have influenced category distribution and introduced measurement bias. Second, because the exact time of the admission decision was not consistently available, an ED LOS of ≥12 h among admitted patients was used as a pragmatic proxy for prolonged boarding. This measure includes the time preceding the admission decision and may therefore misclassify the duration of boarding. Third, median ED occupancy represents an operational proxy for crowding and is not a validated multidimensional crowding index. Established indices such as NEDOCS and EDWIN require variables that were unavailable in our dataset and could not be calculated retrospectively [24]. Fourth, results were not fully consistent across centers, with heterogeneous year-associated differences and, in a few cases, opposite trends. This heterogeneity may reflect unmeasured differences such as changes in staff, hospital capacity, patient case-mix, illness severity, and comorbidities, and might also reflect structural differences in hospital management unexplored by the present study. For instance, the participating ED that developed a more efficient early discharge protocol to nursing care facilities after the pandemic was the only one to experience a consistent reduction in ED LOS and in the number of admitted patients with an ED LOS of ≥12 h. Fifth, the comparison of a single pre-pandemic year with a single post-pandemic year did not capture intermediate temporal trajectories. Consequently, the observed differences cannot be distinguished from secular changes occurring between 2019 and 2024 and should not be interpreted as demonstrating a persistent effect caused by the pandemic. Sixth, since the study was conducted in tertiary referral centers, results may not directly apply to community hospitals. Accordingly, potential solutions of ED overcrowding are likely not “one-size-fits-all”, but should be tailored to local contexts. Finally, the inpatient LOS was available for only two centers in the same region, due to lack of data from the other centers.

## 5. Conclusions

Across four tertiary EDs, annual ED visits were lower in 2024, representing the first full post-pandemic year, mainly because of fewer low-priority and daytime presentations. Despite this reduced input, crowding worsened, with longer door-to-physician time, higher occupancy, more LWBS episodes, longer ED LOS, and longer ED stay among admitted patients. Delays were most evident among patients admitted to general and specialty medical wards. These findings suggest that hospital output constraints may contribute to persistent post-pandemic ED crowding, although their role could not be directly established. Studies incorporating hospital capacity, staffing, discharge processes, and access to post-acute care are needed to identify the underlying mechanisms and evaluate targeted interventions.

## Figures and Tables

**Figure 1:**
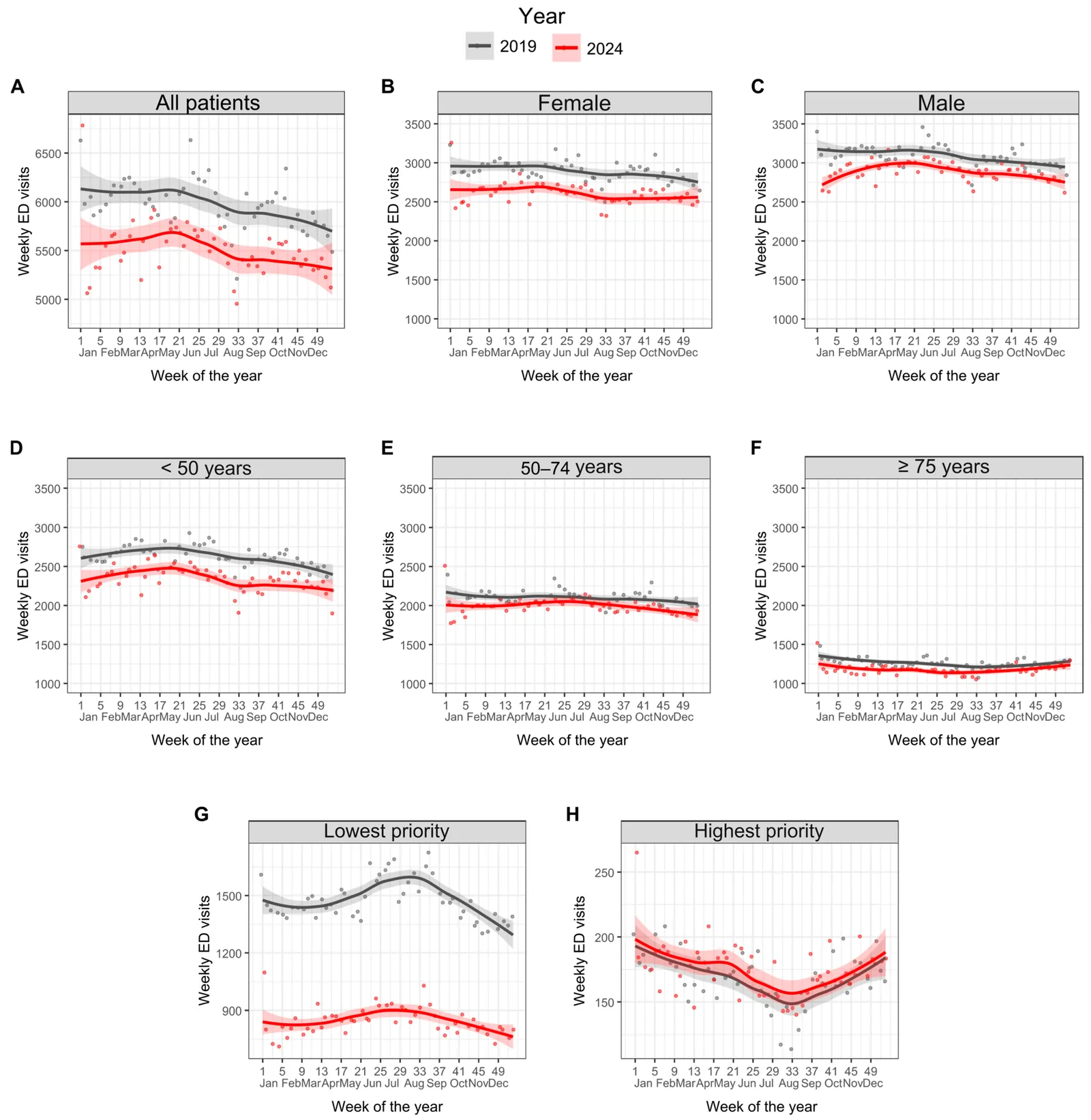
Weekly ED visits in 2019 and 2024 in (**A**) all ED visits, (**B**) female patients, and (**C**) male patients, (**D**–**F**) stratified by three different age groups, and (**G**,**H**) in patients at lowest and highest triage priority.

**Figure 2:**
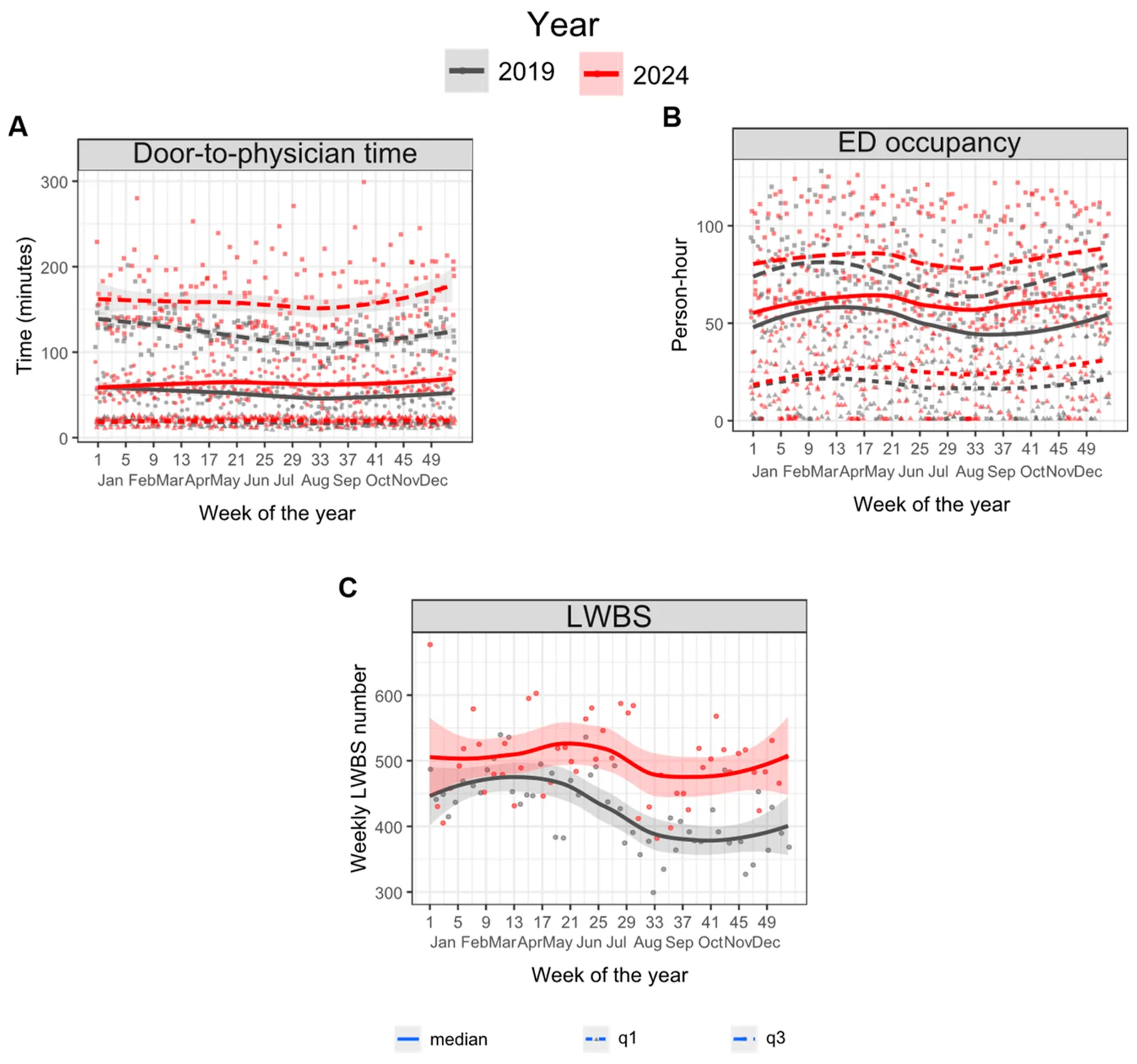
ED throughput and crowding measures in 2019 and 2024. (**A**) Door-to-physician time. (**B**) Ranked median weekly occupancy. (**C**) Number of LWBS visits. Q1 = First quartile, Q3 = third quartile.

**Figure 3:**
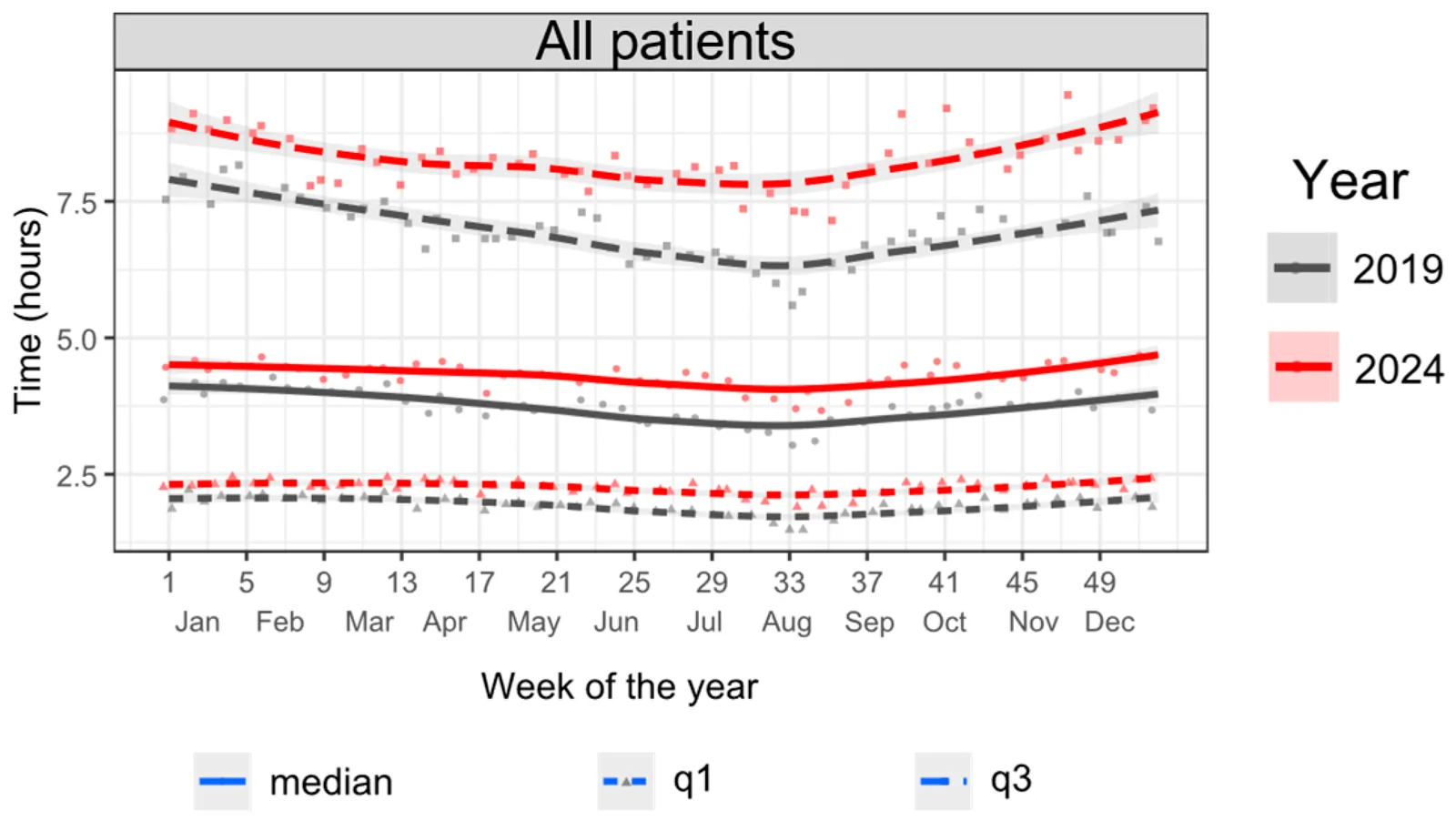
ED length of stay for all ED visits. Q1 = First quartile, Q3 = third quartile.

**Figure 4:**
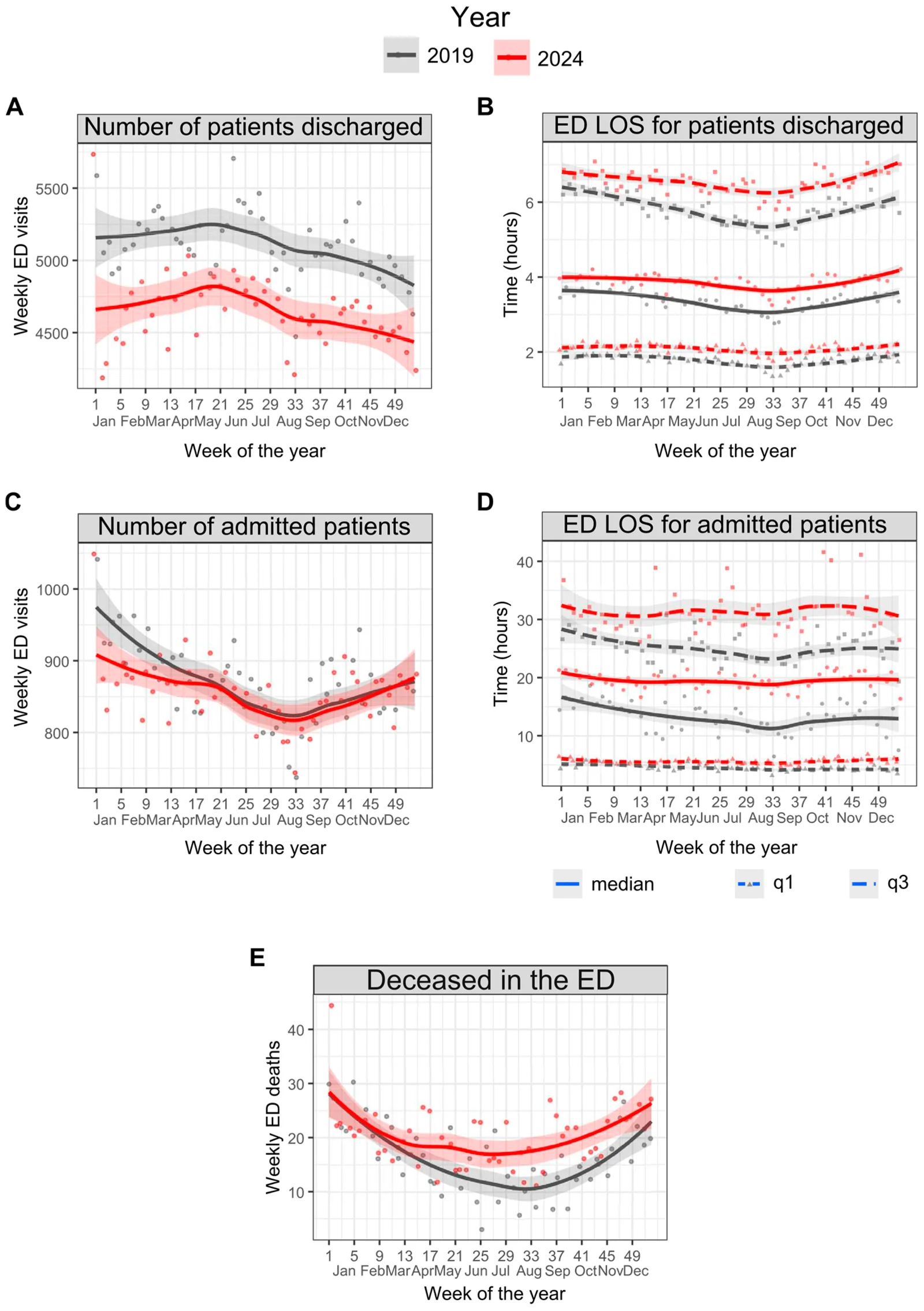
ED output measures according to disposition decision. (**A**) Weekly number of patients discharged home. (**B**) ED length of stay (LOS) for patients discharged home. (**C**) Weekly number of patients admitted to hospital. (**D**) ED LOS for patients admitted to hospital. (**E**) Weekly number of patients deceased in the ED. Q1 = First quartile, Q3 = third quartile.

**Figure 5:**
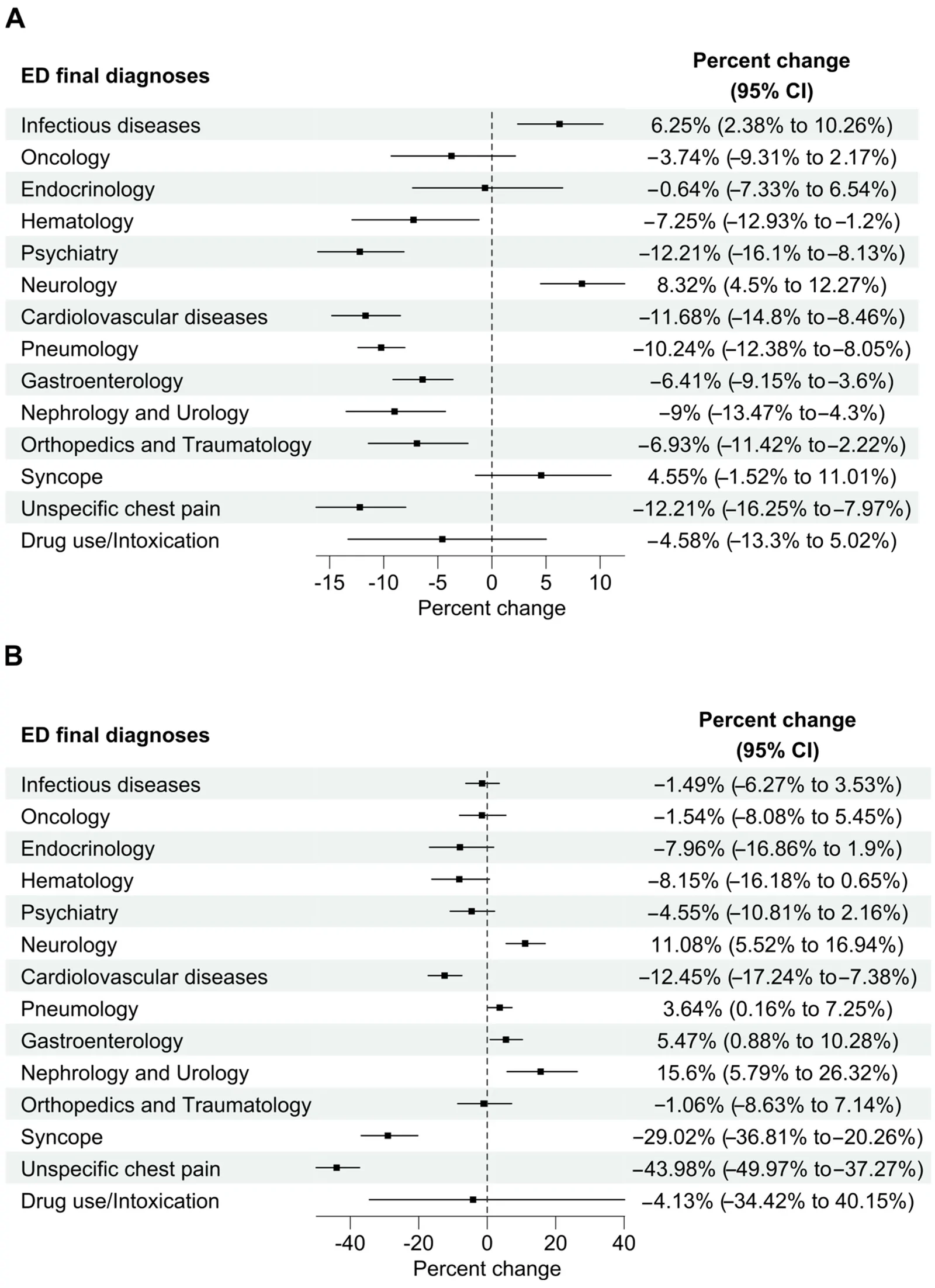
Forest plot showing percentage change in final ED diagnoses (2024 vs. 2019) in (**A**) all ED visits and (**B**) ED visits resulting in hospital admission.

**Table 1:** Changes in input measures between 2024 and 2019.

Outcome	Year 2024	Year 2019	Percentage Change (95% CI)
Total ED visits	286,298	310,900	−5.5% (−9.8 to −1.0)
Sensitivity analysis			
Age: ≥18 years	279,391	302,500	−5.3% (−8.2 to −2.3)
Subgroup analysis			
Males	150,718	160,435	−5.5% (−9.6 to −1.2)
Females	135,580	150,465	−5.5% (−9.4 to −1.4)
Age: <50 years	121,632	136,424	−6.8% (−10.8 to −2.6)
Age: 50–74 years	103,444	108,926	−3.9% (−7.9 to 0.3)
Age: ≥75 years	61,222	65,550	−6.5% (−9.6 to −3.3)
Daytime	207,128	229,046	−6.8% (−9.6 to −4)
Nighttime	79,170	81,854	−2% (−5.2 to 1.3)
Triage codes			
Lowest priority	43,895	76,700	−29.5% (−32.4 to −26.5)
Highest priority	9069	8783	3.1% (−0.8 to 7.2)
Triage symptoms			
Chest pain	25,602	25,729	5.5% (0.4 to 10.9)
Dyspnea	17,255	16,030	7.3% (1.4 to 13.5)
Abdominal pain	30,724	30,103	4.7% (−0.2 to 9.8)
Psychiatric symptoms	7427	5044	38.9% (30.5 to 48)
Trauma	85,955	91,034	0.5% (−4.8 to 6.2)
Intoxication/Drug use	4725	4255	10.7% (3.9 to 17.9)

**Table 2:** Output measures (times are reported as median). LOS: Length of stay.

Outcome	Year 2024	Year 2019	Percentage Change (95% CI)
ED LOS			
Total	4 h 27 min	3 h 48 min	15.1% (14.4 to 15.7)
Discharged home	3 h 54 min	3 h 23 min	6.0% (5.3 to 6.6)
Admitted to hospital	19 h 21 min	13 h 17 min	29% (27.3 to 30.8)
Disposition decision			
Home	210,369	238,736	−6.6% (−10.4 to −2.6)
Ward	44,505	45,313	−4.8% (−9.1 to −0.3)
Admitted patients with ED LOS of ≥12 h	27,934	23,922	13.8% (10.3 to 17.3)
Deceased in the ED	1053 (0.4%)	843 (0.3%)	23.3% (11.8 to 35.9)

**Table 3:** Variables related to hospital admission.

Outcome	Year 2024	Year 2019	Percentage Change (95% CI)
General and specialty medicine			
Number	27,469	26,765	3.2% (−0.01 to 6.5)
ED LOS	24 h 36 min	18 h 42 min	35.8% (33.9 to 37.7)
General and specialty surgery			
Number	9075	10,151	−9.8% (−13.6 to −5.8)
ED LOS	7 h 38 min	7 h 31 min	3.1% (0.03 to 6.3)
Critical wards			
Number	6612	7152	−7.3% (−11.3 to −3.1)
ED LOS	5 h 24 min	6 h 12 min	−7.5% (−10.8 to −4.1)
Psychiatry			
Number	1294	1245	4.7% (−3.2 to 13.2)
ED LOS	2 h 31 min	1 h 53 min	39.5% (26.5 to 53.9)

## Data Availability

Data are not publicly available due to legal restrictions.

## Supplementary Materials

The following supporting information can be downloaded at https://www.mdpi.com/article/10.3390/jcm15197450/s1. Table S1: Diagnosis grouping based on ICD-9-CM codes. Table S2: Bed capacity in general/specialty medical wards and critical care units at participating centers in 2024 and 2019. Table S3: Inpatient length of stay (median values) in 2024 and 2019 for CDSS and OSGB. Figure S1: Total ED visits in 2019 and 2024 stratified by center. Figure S2: ED visits by time of the day. “Night” was defined as time of registration between 8 pm and 8 am. Figure S3: ED visits by main triage symptoms. Figure S4: Admitted patients with an ED LOS of ≥12h. Figure S5: ED LOS among admitted patients stratified by destination ward. Figure S6: Adjusted percentage change (2024 vs 2019) in major outcomes by center.

## Abbreviations

The following abbreviations are used in this manuscript:

ED	Emergency department
LOS	Length of stay
LWBS	Left without being seen
