# Supplementary material for: Emergency Department Overcrowding Despite Lower Post-Pandemic Volumes: A Multicenter Study
Source: J Clin Med. 2026 Sep 24;15(19):7450. doi: 10.3390/jcm15197450 (PMC13665870; doi:10.3390/jcm15197450)

SUPPLEMENTARY DATA

Emergency department overcrowding despite lower post-pandemic volumes: a multicenter study

Supplemental Table S1. Diagnosis grouping based on ICD-9-CM codes. 2

Supplemental Table S2. Bed capacity in general/specialty medical wards and critical care units at participating centers in 2024 and 2019.. 3

Supplemental Table S3. Inpatient length of stay (median values) in 2024 and 2019 for CDSS and OSGB. 4

Supplemental Figure S1. Total ED visits in 2019 and 2024 stratified by center. 5

Supplemental Figure S2. ED visits by time of the day. "Night" was defined as time of registration between 8 pm and 8 am. 6

Supplemental Figure S3. ED visits by main triage symptoms. 7

Supplemental Figure S4. Admitted patients with an ED LOS of ≥12 h. 8

Supplemental Figure S5. ED LOS among admitted patients stratified by destination ward. 9

Supplemental Figure S6. Adjusted percent change (2024 vs 2019) in major outcomes by center. P-value for interaction was statistically significant for all outcomes (P<0.001 for all variables except for patient deceased in the ED: P = 0.03). 10

Supplemental Table S1. Diagnosis grouping based on ICD-9-CM codes.

**Diagnosis group**	**ICD-9-CM codes**
Infectious diseases	001-139; 460-465; 480-488; 590.1-590.9; 785.52; 780.6; 995.90-995.99
Oncology	140-239
Endocrinology	240-279
Hematology	280-289
Psychiatry	290-302; 306-319
Neurology	320-359; 430-439; 780.3; 781-782.0; 784.0
Cardiology	390-429; 440-459; 441.0-441.99; 785.0-785.51; 997.1; 997.91
Pneumology	490-519; 466; 786.0-786.49
Gastroenterology	520-579; 787.0-787.99; 789.0-789.99
Urology	580-590.0; 590.9-599; 788.0-788.99
Orthopedics and trauma	800-959.9
Drug use/Intoxication	303-305.93; 965.0-965.99; 980.0-980.99
Syncope	780.2
Unspecific chest pain	786.50-786.59

Supplemental Table S2. Bed capacity in general/specialty medical wards and critical care units at participating centers in 2024 and 2019.

Ward type	2024	2019	Absolute change
General and specialty medicine	1,167	1,188	-21
Turin (CDSS)	387	415	-28
Rome (PUG)	430	410	+20
Milan (OMP)	204	204	0
Turin (OSGB)	146	159	-13
Critical care	304	296	+8
Turin (CDSS)	102	102	0
Rome (PUG)	110	110	0
Milan (OMP)	52	44	+8
Turin (OSGB)	40	40	0
Total (medicine + critical care)	1,471	1,484	-13

Legend. Absolute change was calculated as the 2024 value minus the 2019 value. Critical care was broadly defined to include general and specialty intensive care units, coronary care units, stroke units, semi-intensive or high-dependency units, and emergency medicine units. CDSS, Città della Salute e della Scienza di Torino; OMP, Ospedale Maggiore Policlinico; OSGB, Ospedale San Giovanni Bosco; PUG, Policlinico Universitario Gemelli.

Supplemental Table S3. Inpatient length of stay (median values) in 2024 and 2019 for CDSS and OSGB.

Ward	2024	2019	
	days	days	Percent change (95% CI)
All wards	9.1	8.3	5.9% (4.0 to 7.7)
General and specialty medicine	10.0	9.1	5.9% (3.6 to 8.2)
Critical wards	8.22	8.33	1.7% (-3.1 to 6.7)
Psychiatry	9.8	10.8	-23.1% (-29.1 to -16.7)

Supplemental Figure S1. Total ED visits in 2019 and 2024 stratified by center.

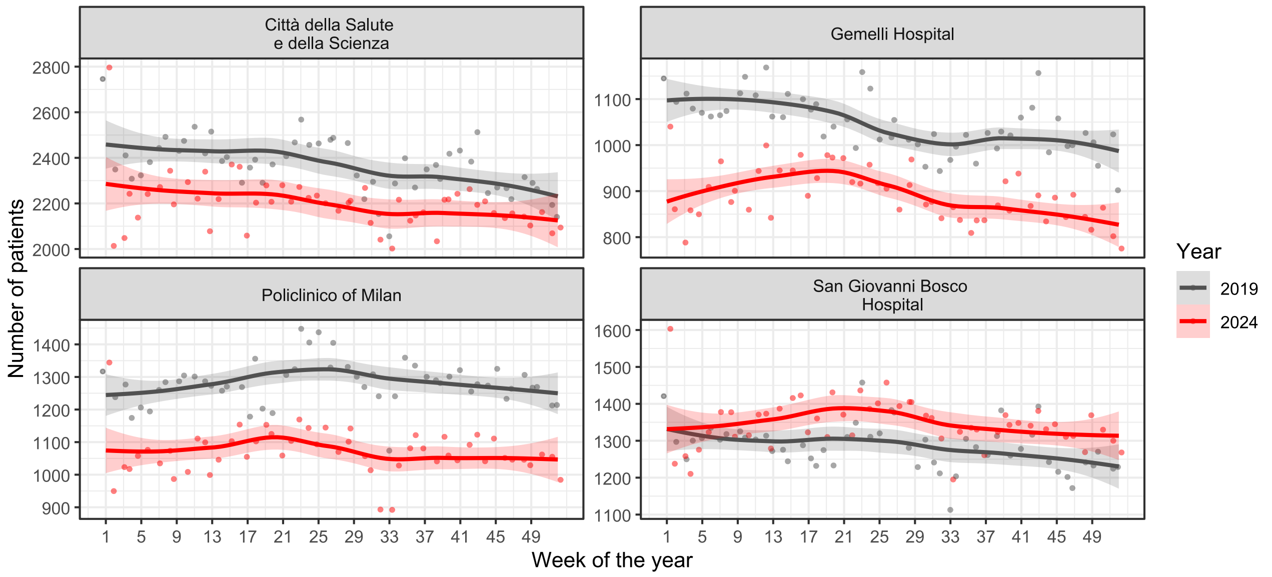

Supplemental Figure S2. ED visits by time of the day. "Night" was defined as time of registration between 8 pm and 8 am.

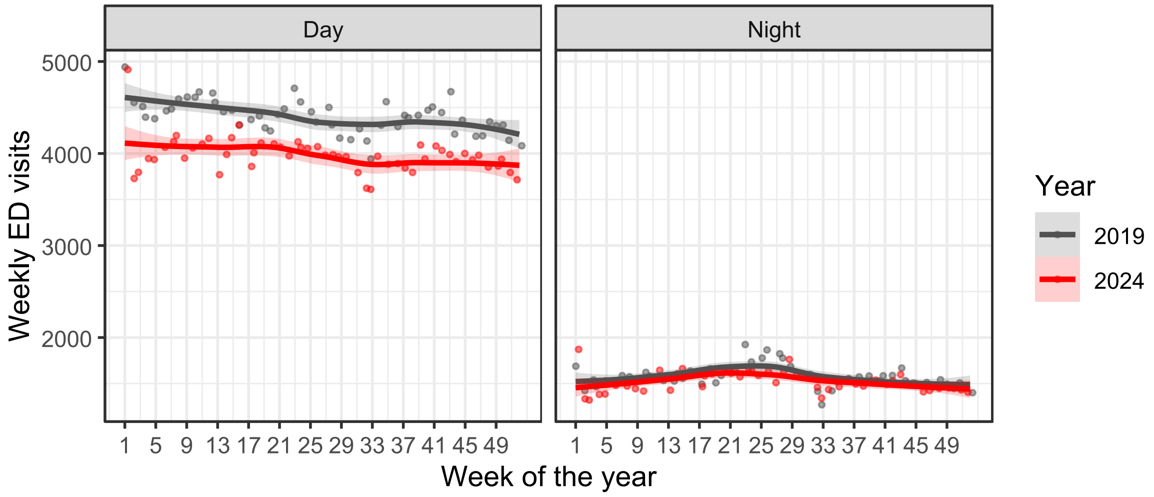

Supplemental Figure S3. ED visits by main triage symptoms.

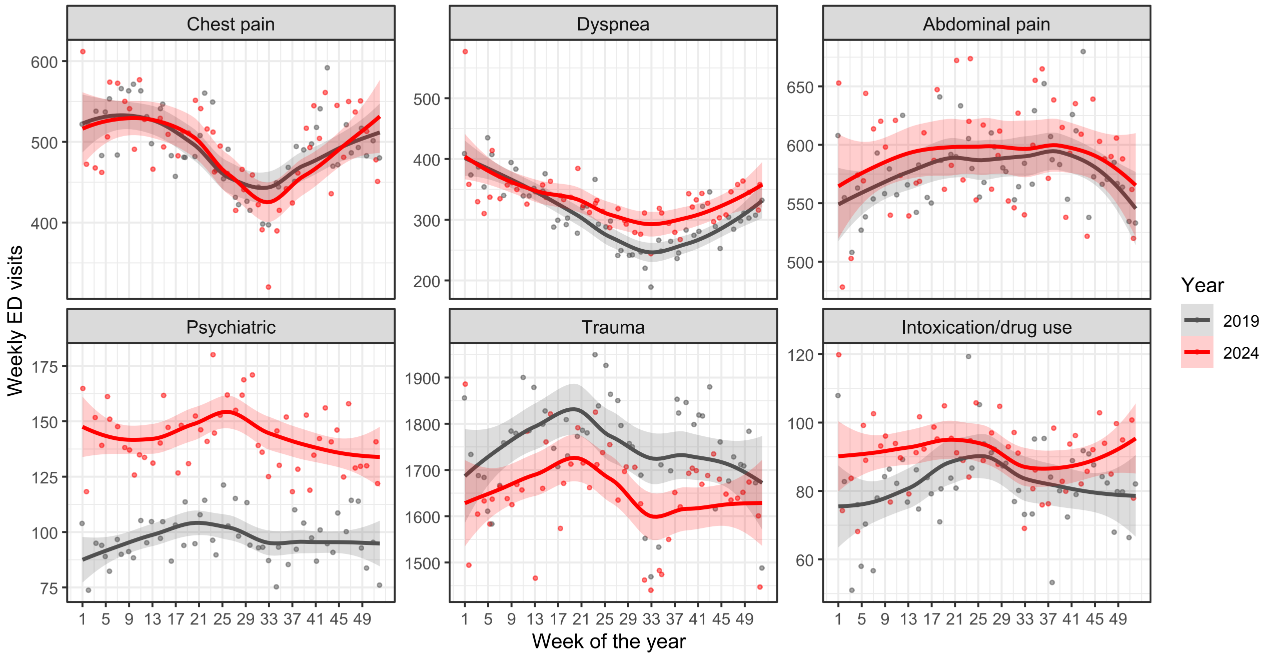

Supplemental Figure S4. Admitted patients with an ED LOS of ≥12 h.

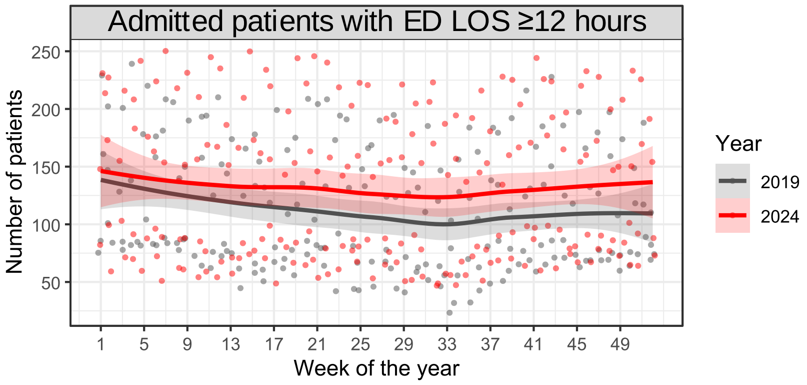

Supplemental Figure S5. ED LOS among admitted patients stratified by destination ward.

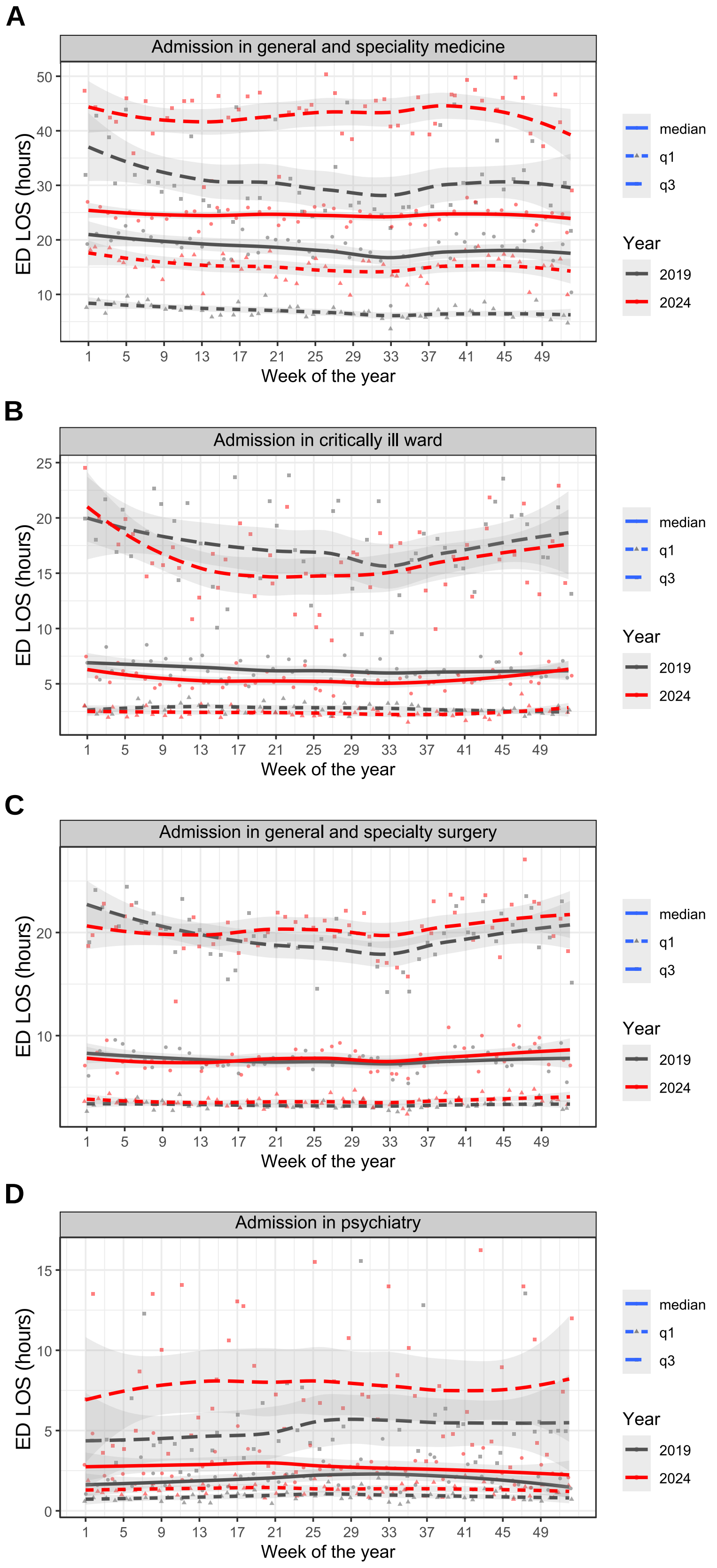

Supplemental Figure S6. Adjusted percent change (2024 vs 2019) in major outcomes by center. P-value for interaction was statistically significant for all outcomes (P<0.001 for all variables except for patient deceased in the ED: P = 0.03).

Outcome	center	Percent change (95%CI)
**All patients**		
	Città della Salute e della Scienza	-7.04% (-9.67 to -4.33)
	Gemelli Hospital	-14.95% (-17.35 to -12.49)
	Policlinico of Milan	-15.63% (-18.23 to -12.94)
	San Giovanni Bosco Hospital	4.88% (0.71 to 9.21)
**Patients admitted to hospital**		
	Città della Salute e della Scienza	-12.11% (-15.97 to -8.08)
	Gemelli Hospital	1.76% (-2.36 to 6.07)
	Policlinico of Milan	11.32% (5.53 to 17.44)
	San Giovanni Bosco Hospital	3.59% (-2.71 to 10.3)
**Patients discharged home**		
	Città della Salute e della Scienza	-5.81% (-8.93 to -2.59)
	Gemelli Hospital	-30.63% (-32.6 to -28.59)
	Policlinico of Milan	-24.79% (-27.19 to -22.31)
	San Giovanni Bosco Hospital	3.86% (-0.23 to 8.12)
**Patients who left without being seen**		
	Città della Salute e della Scienza	-2.41% (-6.7 to 2.07)
	Gemelli Hospital	42.92% (34.54 to 51.81)
	Policlinico of Milan	34.15% (25.17 to 43.77)
	San Giovanni Bosco Hospital	12.19% (5.98 to 18.76)
**Patients who died in the ED**		
	Città della Salute e della Scienza	41.66% (11.56 to 79.88)
	Gemelli Hospital	23.27% (2.93 to 47.64)
	Policlinico of Milan	-2.13% (-19.72 to 19.31)
	San Giovanni Bosco Hospital	37.62% (17.62 to 61.01)
**Door-to-physician time**		
	Città della Salute e della Scienza	5.24% (4.3 to 6.19)
	Gemelli Hospital	92.24% (89.35 to 95.17)
	Policlinico of Milan	26.62% (25.01 to 28.24)
	San Giovanni Bosco Hospital	5.47% (4.18 to 6.79)
**ED LOS for all patients**		
	Città della Salute e della Scienza	9.37% (8.48 to 10.28)
	Gemelli Hospital	47.95% (45.82 to 50.11)
	Policlinico of Milan	44.16% (42.37 to 45.98)
	San Giovanni Bosco Hospital	-18.67% (-19.66 to -17.67)
**ED LOS for discharged patients**		
	Città della Salute e della Scienza	3.86% (2.98 to 4.75)
	Gemelli Hospital	16.86% (14.77 to 18.99)
	Policlinico of Milan	46.94% (44.91 to 48.99)
	San Giovanni Bosco Hospital	-12.05% (-13.17 to -10.92)
**ED LOS for hospitalized patients**		
	Città della Salute e della Scienza	33.02% (30.34 to 35.75)
	Gemelli Hospital	69.56% (65.59 to 73.63)
	Policlinico of Milan	31.43% (27.16 to 35.84)
	San Giovanni Bosco Hospital	-43.65% (-45.62 to -41.62)

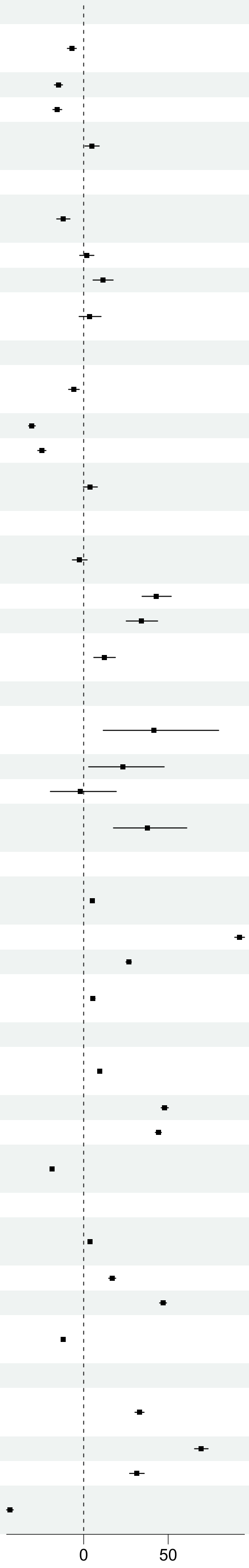

Supplement: Supplementary file 1 [file jcm-15-07450-s001.zip › jcm-4527217-supplementary.pdf]
